# La investigación médica en tiempos de COVID-19

**DOI:** 10.47487/apcyccv.v1i2.61

**Published:** 2020-06-29

**Authors:** Manuel Chacón-Díaz

**Affiliations:** 1 Servicio de Cardiología Clínica - Instituto Nacional Cardiovascular - INCOR EsSalud. Lima, Perú. Servicio de Cardiología Clínica Instituto Nacional Cardiovascular - INCOR EsSalud Lima Perú; 2 Editor general APCyCCV

El 6 de marzo del 2020, se detectó en el Perú el primer caso de la enfermedad COVID-19; cien días después, más de un cuarto de millón de personas han sido reportadas como contagiadas y más de ocho mil como fallecidas;^1^^)^ aunque la verdadera magnitud de la pandemia definitivamente debe tener resultados mucho más abultados.

Los efectos de la infección a nivel cardiovascular son cada vez más reportados, encontrándonos en la actualidad ante una entidad que además de comprometer el pulmón, genera directa o indirectamente daño a nivel cardíaco, desde miocarditis, injuria miocárdica, isquemia y necrosis, fenómenos trombo-embólicos y también episodios de arritmias potencialmente letales, muchas veces producidas por la medicación que administramos. ^(^^2^^)^

En los últimos meses, mucha literatura se ha escrito a nivel mundial sobre la presentación, fisiopatología y posibles estrategias de tratamiento. Así, hemos podido observar desde publicaciones acerca de una novedosa asociación de antimaláricos con macrólidos^3^^)^ (que se hizo muy popular a nivel global como la esperanza de tratamiento contra el virus a pesar de su muy cuestionada validez científica, y ha sido la base del tratamiento de soporte de la enfermedad en muchos establecimientos de salud en nuestro país), hasta publicaciones acerca de antivirales^4^^)^ que no han demostrado un efecto beneficioso ante el SARS-CoV-2 o sus hallazgos son cuestionables, ^(^^5^^)^ antiparasitarios con efecto in vitro, ^(^^6^^)^ corticoesteroides, inhibidores del interleukina ^6^, ^(^^7^^)^ etc., que se han administrado a muchos pacientes.

Debido a ello, nos preguntamos: ¿Por qué, a pesar de que hace muchos años, practicamos la medicina basada en la evidencia científica, no hemos hecho caso a esta y hemos ofrecido como tratamiento lo primero - aparentemente alentador - que ha salido en la literatura, sin reparar en la metodología científica, en la importancia del grupo control, de la aleatorización (randomización) de los individuos a un determinado tratamiento, de la evaluación estadística, de la evaluación por pares y demás características que hacen de un ensayo clínico valedero?, ¿será que en tiempo de pandemia el rigor científico no cuenta mucho?, o ¿que nos aferramos a una esperanza que nos “venden” en la literatura para disminuir la morbimortalidad de una enfermedad nueva a la que apenas estamos comenzando a entender? Muchos colegas han alegado a favor del uso de dichos fármacos porque “no hay alternativas” o “no hay estudios más grandes y adecuados que nos den otra alternativa” o simplemente porque “la FDA lo aprobó”. Inclusive, revistas de gran renombre han caído en el error de publicar artículos aparentemente con datos falsos, que no han hecho más que generar dudas en la comunidad médica global. Obviamente las revistas confían en los datos que los investigadores envían, ya que parte esencial de este flujo en la investigación es la ética de los autores.

Es ante estos hechos, que no debemos perder el horizonte del método científico como el proceso generador de nuevas evidencias médicas. “*Primun non nocere*” ha sido nuestro guía desde que comenzamos la práctica profesional en salud, y en estos momentos, donde la humanidad está golpeada por esta pandemia no debe ser la excepción; es más, deberíamos ser parte de estudios clínicos bien estructurados con lo que estaríamos sumando al conocimiento de esta y otras enfermedades; asimismo, aplicar siempre la lectura crítica a profundidad cuando revisamos nueva literatura y más aún en situaciones donde la salud global está en riesgo.

**Figura 1:**
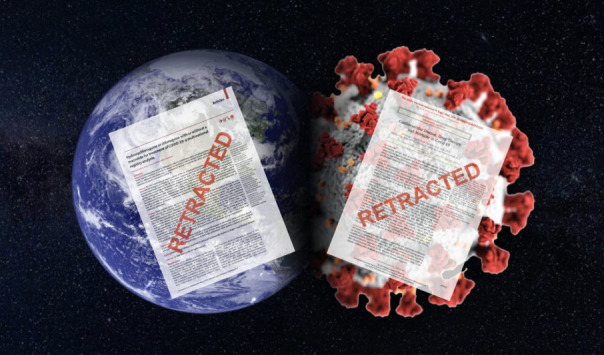
2 revistas médicas de prestigio internacional han retirado artículos relacionados al tratamiento del COVID-19. Adaptado de Tumlinson R. Op-ed | The need to dream in darkness - SpaceNews.com [Internet]. SpaceNews.com. 2020 [citado 25 de junio de 2020]. Disponible en: https://spacenews.com/op-ed-the-need-to-dream-in-darkness/ Arte original de Robin McDowall.

En este contexto, la segunda edición de Archivos Peruanos de Cardiología y Cirugía Cardiovascular está dedicada en parte a las primeras experiencias nacionales acerca del impacto de la pandemia por COVID 19 en el ámbito de la salud cardiovascular; por ejemplo: Custodio y colaboradores, en representación de los investigadores del registro PERSTEMI-2, presentan el artículo **“Impacto de la pandemia por Covid-19 en la atención del infarto de miocardio ST elevado en el Perú”,** donde nos hablan sobre la disminución de atenciones por esta enfermedad durante la pandemia, algo visto a nivel mundial y reportado por muchos autores, discutiendo las probables causas de este hallazgo. Yarahuaman y colaboradores presentan el artículo: **“Infección por SARS-CoV-2 en pacientes con trasplante cardíaco, reporte de casos”**, donde describen dos casos de pacientes trasplantados de corazón que sufren complicaciones por COVID-19 que los lleva a hospitalizarse, y finalmente la Sociedad Peruana de Hipertensión Arterial, por intermedio del Dr. Alfonso Bryce, en su artículo “**El tratamiento con bloqueadores del sistema Renina-Angiotensina-Aldosterona no debe suspenderse tras la infección por SARS-CoV-2”** nos recuerda la asociación de la enfermedad con la hipertensión arterial y su tratamiento.

Además, en este número tenemos la participación de colegas latinoamericanos, así Saldarriaga y colaboradores, desde Medellín - Colombia, nos presentan un detallado artículo de revisión titulado **“Falla cardíaca con fracción de eyección preservada: un problema de la cardiología contemporánea”,** y Cabrera y colaboradores desde México nos presentan un caso pediátrico inusual en su artículo: **“Revascularización coronaria en enfermedad de Kawasaki”**; a quienes agradecemos por confiar en nuestra revista para sus publicaciones, lo que nos compromete a mejorar cada vez más en el proceso editorial, para lograr el impacto que poco a poco esperamos tenga esta revista en la comunidad cardiovascular latinoamericana.

Por otro lado, a partir de este número incluimos en nuestra página web, una sección de trazados electrocardiográficos mensuales que serán descritos por expertos electrofisiólogos, con los “*tips*” para su diagnóstico y el link hacia el blog de ecocardiografía del Dr. Mario Vargas, a quienes agradecemos por unirse a la familia de la revista.

Los miembros del consejo editorial de Archivos Peruanos de Cardiología y Cirugía Cardiovascular esperamos que los artículos de este número sean de su interés.
